# Certain Grain Foods Can Be Meaningful Contributors to Nutrient Density in the Diets of U.S. Children and Adolescents: Data from the National Health and Nutrition Examination Survey, 2009–2012

**DOI:** 10.3390/nu9020160

**Published:** 2017-02-20

**Authors:** Yanni Papanikolaou, Victor L. Fulgoni

**Affiliations:** 1 Nutritional Strategies, 59 Marriott Place, Paris, ON N3L 0A3, Canada; 2 Nutrition Impact, 9725 D Drive North, Battle Creek, MI 49014, USA; vic3rd@aol.com

**Keywords:** NHANES, energy, nutrients, children, grains

## Abstract

Grain foods may play an important role in delivering nutrients to the diet of children and adolescents. The present study determined grain food sources of energy/nutrients in U.S. children and adolescents using data from the National Health and Nutrition Examination Survey, 2009–2012. Analyses of grain food sources were conducted using a 24-h recall in participants 2–18 years old (*N* = 6109). Sources of nutrients contained in grain foods were determined using U.S. Department of Agriculture nutrient composition databases and excluded mixed dishes. Mean energy and nutrient intakes from the total diet and from various grain foods were adjusted for the sample design using appropriate weights. All grains provided 14% ± 0.2% kcal/day (263 ± 5 kcal/day), 22.5% ± 0.3% (3 ± 0.1 g/day) dietary fiber, 39.3% ± 0.5% (238 ± 7 dietary folate equivalents (DFE)/day) folate and 34.9% ± 0.5% (5.6 ± 0.1 mg/day) iron in the total diet in children and adolescents. The current analyses showed that certain grain foods, in particular breads, rolls and tortillas, ready-to-eat cereals and quick breads and bread products, are meaningful contributors of folate, iron, thiamin, niacin and dietary fiber, a nutrient of public health concern as outlined by the 2015–2020 Dietary Guidelines for Americans. Thus, specific grain foods contribute to nutrient density and have the potential to increase the consumption of several under-consumed nutrients in children and adolescents.

## 1. Introduction

Mandatory folic acid fortification, commencing in 1998 by the Food and Drug Administration [1], highlighted the importance of grains in the delivery of essential nutrients. Specific grain foods becoming leading sources for folate with breads, rolls and crackers considered a leading contributor of total folate to the U.S. diet representing 16% of total intake [2]. Data from the National Health and Nutrition Examination Survey (NHANES), 2003–2006, showed fortification of grain foods provides nutrient adequacy for U.S. children and adolescents, without concern for excessive intakes for most vitamins and minerals [3]. Recently, the 2015–2020 Dietary Guidelines for Americans (2015–2020 DGA) recommended all individuals two years of age and older meet nutrient requirements through foods as part of a nutrient-dense diet; however, many individuals fall short in several nutrients [4], resulting in the declaration that several nutrients are under-consumed relative to the estimated average requirement (EAR; the average daily nutrient intake level estimated to meet the requirements of half of the healthy individuals in a group) or adequate intake levels established by the Institute of Medicine [5]. The 2015–2020 DGA characterized under-consumed nutrients as shortfall nutrients and included vitamins A, D, E, C, folate, calcium, magnesium, fiber, potassium and iron in specific populations. Similar to the preceding policy and scientific advisory committee reports [6,7], the 2015–2020 DGA continues to highlight calcium, potassium, vitamin D and fiber as shortfall nutrients of public health concern’ in Americans, including children, since under-consumption of these nutrients has been associated with adverse health outcomes [4]. The nutrient shortfall is further exacerbated in specific American subpopulations, including lower-income households and children of different ethnicities relative to dietary recommendations [8,9,10,11,12,13]. An analysis of the National Health and Nutrition Examination Survey (NHANES) 2007–2010 analysis identified significantly lower usual nutrient intakes in both non-Hispanic black children and adults when compared to their non-Hispanic white counterparts, such that non-Hispanic black children had lower intakes of calcium, phosphorus, magnesium, vitamins A and D [14].

Dietary recommendations advocate for greater whole grain consumption while limiting refined grains in the diet. While certain grain food products contribute to nutrients to limit in the diet, including added sugar and saturated fat [15,16], grain foods as a category, including refined grains, also provide a range of positive nutrients, including dietary fiber, iron, magnesium and B vitamins (thiamin, riboflavin, niacin and folate). Previous work using data from NHANES 2003–2006 showed that grain foods provided meaningful contributions for dietary fiber, calcium, magnesium, iron, thiamin, riboflavin, niacin and folic acid. Further, data from NHANES 2003–2006 in Americans aged ≥2 years old identified that significant amounts of vitamins A, B6, B12, C and D, as well as thiamin, riboflavin, niacin, folate and iron were from enriched and/or fortified foods with the researchers concluding that without enrichment and/or fortification, nutrient shortfalls are further exacerbated [17].

Taking into account the importance of nutrient recommendations for American children and adolescents, the objective of the present analyses was to determine sources of energy and nutrients in commonly-consumed grain foods in U.S. children and adolescents using data from the NHANES, 2009–2012.

## 2. Experimental Section

The NHANES is a nationally-representative, cross-sectional survey of U.S. non-institutionalized, civilian residents. NHANES data are collected by the National Center for Health Statistics of the Centers for Disease Control and Prevention. Written informed consent was obtained for all participants or proxies, and the survey protocol was approved by the Research Ethics Review Board at the National Center for Health Statistics. Data from two NHANES datasets (2009–2010; 2011–2012) were combined for the present analyses in children and adolescents [18,19] for age groups as established by the Food and Nutrition Board, Institute of Medicine for the Dietary Reference Intakes (i.e., 1–3, 4–8, 9–13, 14–18 and 2–18 years old; [20]). Nutrient intake data for NHANES 2009–2012 are from the United States Department of Agriculture (USDA) Food and Nutrient Database for Dietary Studies (FNDDS) 5.0 [21]. FNDDS is a database that provides the nutrient values for foods and beverages reported in What We Eat in America (WWEIA), the dietary intake component of NHANES. The WWEIA Food Categories provide an application to analyze food and beverages as consumed in the American diet. For the current analyses, each of the grain foods reported in WWEIA/NHANES is placed in one of the mutually-exclusive food categories by linking each food code contained in FNDDS to one WWEIA category [22]. This food code classification scheme includes approximately 150 unique food categories. Mixed grain dishes were separated from the main grains group and are not included in the current analyses.

In the present analyses, the combined sample included 6109 male and female participants, 2–18 years of age, who had reliable and complete 24-h dietary intake data from WWEIA.

### 2.1. Methods and Statistical Analysis

All statistical analyses were performed using SAS software (Version 9.2, SAS Institute, Cary, NC, USA). SAS PROC SQL was used to create and prepare data for analyses. SAS PROC SURVEYMEANS was used for all statistical calculations, including means and percentages. Survey weights were used to generate nationally-representative estimates for U.S. children and adolescents, which were adjusted for the complex sample design of NHANES. Mean and standard errors of energy, macro- and micro-nutrients for the daily total diet and from food groups were determined. The mean percentage of energy and nutrients provided by each grain food group was also determined. Meaningful sources of energy and nutrients from the overall grain category and the various sub-categories of grain products were defined as an energy or nutrient contribution of ≥10 percent of the total diet per day. A red solid line was incorporated in all figures to indicate the percentage of energy provided by the food category of interest. Those nutrients above the red line show that the food category provides a higher percentage in the diet relative to energy, thus indicating nutrient density.

## 3. Results

In addition to an all grains category, six main grain food groups were identified from NHANES and categorized by mean gram weight consumption in the total diet (Table 1).

The various grain food sources of energy and nutrients for various age groups are shown in Figure 1, Figure 2, Figure 3, Figure 4, Figure 5, Figure 6, Figure 7, Figure 8, Figure 9, Figure 10, Figure 11, Figure 12, Figure 13, Figure 14, Figure 15, Figure 16, Figure 17, Figure 18, Figure 19, Figure 20, Figure 21, Figure 22, Figure 23, Figure 24, Figure 25, Figure 26, Figure 27, Figure 28, Figure 29 and Figure 30. Age ranges were chosen as established by the Food and Nutrition Board, Institute of Medicine for Dietary Reference Intakes (i.e., 1–3, 4–8, 9–13 and 14–18 years old; [23]). Energy and twenty-four nutrients are listed from all grain foods and various subsets within commonly-consumed grain foods (i.e., breads, rolls, tortillas; ready-to-eat cereals; cooked grains; quick breads; and sweet bakery products), which include nutrients to limit and encourage in the daily diet as outlined by dietary guidance [4]. A red solid line was incorporated in all figures to indicate the percentage of energy provided by the grain food category of interest. For nutrients above the red line, the grain food category can be considered as contributing to nutrient density.

### 3.1. All Grain Foods: Sources of Energy and Nutrients

**Figure 1:**
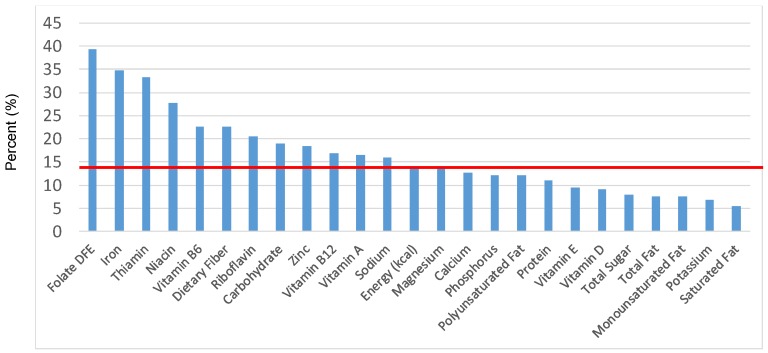
Grain foods sources of energy and nutrients for children and adolescents, 2–18 years old (*N* = 6109, daily intake data): DFE = Dietary Folate Equivalents; solid red line represents percentage of energy (kcal) provided in the diet relative to nutrients—nutrients above the red line show the grain food’s contribution to nutrient density.

The percentage of total energy and nutrients provided from the total grain food category, excluding mixed grain dishes, can be seen in Figure 1. While approximately 16% of sodium, 14% of energy, 8% of total sugar, 7.6% of total fat and 5.4% of saturated fat were sourced from grains in the daily diet, grain foods also provided approximately nearly a quarter of total dietary fiber per day (22.5%). Grain foods also provided approximately 39.3% of dietary folate, 34.8% of iron, 16.4% of vitamin A and 13.7% of magnesium on a daily basis, all nutrients identified as shortfall nutrients by the 2015–2020 DGA. Thus, considering all grains only make up 5.18% of the mean gram weight of the total diet (see Table 1), a meaningful nutrient density profile is apparent for 14% of total energy in the diet, in addition to delivering several 2015–2020 DGA shortfall nutrients.

### 3.2. Breads, Rolls and Tortillas: Sources of Energy and Nutrients

**Figure 6:**
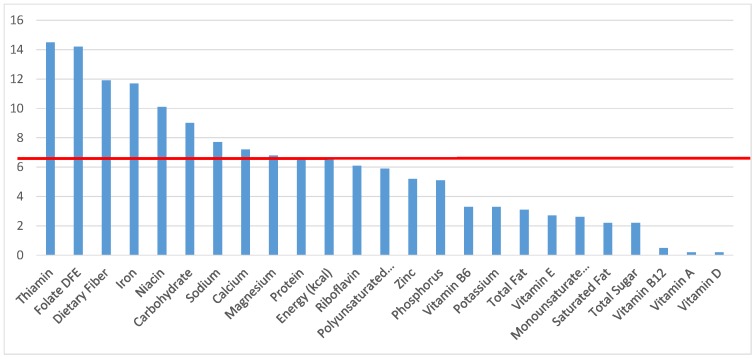
Breads, Rolls and tortilla as sources of energy and nutrients for children and adolescents 2–18 years old (*N* = 6109, daily intake data): DFE = Dietary Folate Equivalents; solid red line represents percentage of energy (kcal) provided in the diet relative to nutrients—nutrients above the red line show the grain food’s contribution to nutrient density.

The percentage of total dietary intake of energy and nutrients provided from the bread, rolls and tortilla category, excluding mixed grain dishes, can be seen in Figure 6. When considering nutrients to limit, breads, rolls and tortillas provided 7.7% of sodium, 2.2% of total sugar, 3.1% of total fat and 2.2% of saturated fat. Breads, rolls and tortillas contributed 6.6% of total energy in the American diet of children and adolescents daily and approximately 12% total dietary fiber per day. Breads, rolls and tortillas also provided approximately 14.2% of folate, 11.7% of iron, 7.2% of calcium and 6.8% of magnesium on a daily basis, all nutrients identified as shortfall nutrients by the 2015–2020 Dietary Guidelines for Americans.

### 3.3. Ready-to-Eat Cereals: Sources of Energy and Nutrients

**Figure 11:**
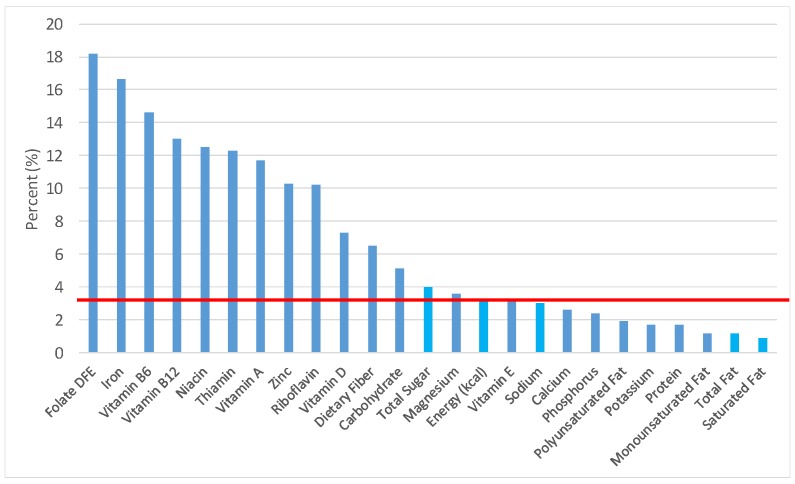
Ready-to-eat cereals as sources of energy and nutrients for children and adolescents (*N* = 6109, daily intake data): DFE = Dietary Folate Equivalents; solid red line represents percentage of energy (kcal) provided in the diet relative to nutrients—nutrients above the red line show the grain food’s contribution to nutrient density.

The percentage of total dietary intake of energy and nutrients provided from ready-to-eat cereals can be seen in Figure 11. When considering nutrients to limit, the ready-to-eat cereals category provided 3.0% of sodium, 4.0% of total sugar, 1.2% of total fat and 0.9% of saturated fat in the daily diet. While ready-to-eat cereals contributed limited calories (58 ± 2.3 kcal per day), such that the category provided 3.3% of total energy to the diet of children and adolescents daily, ready-to-eat cereals provided 6.5% of total dietary fiber per day. Ready-to-eat cereals also provided approximately 18.2% of dietary folate, 16.6% of iron, 12.3% of thiamin, 13.0% of vitamin B12, 11.7% of vitamin A, 12.5% of niacin, 10.3% of zinc and 7.3% of vitamin D on a daily basis.

### 3.4. Cooked Grains: Sources of Energy and Nutrients

**Figure 16:**
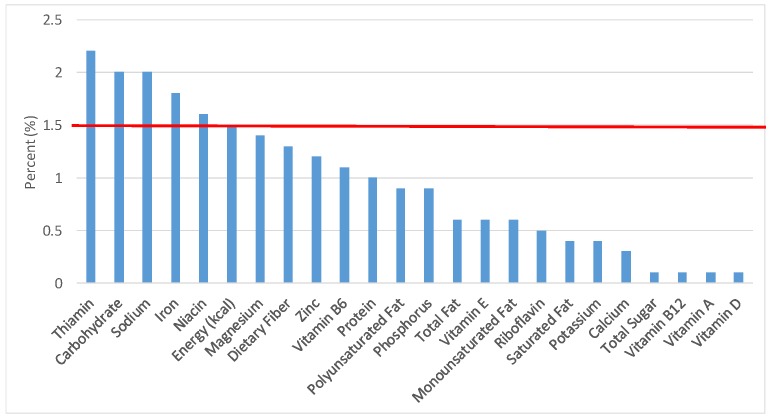
Cooked grains as sources of energy and nutrients for children and adolescents 2–18 years old (*N* = 6109, daily intake data): DFE = Dietary Folate Equivalents; solid red line represents percentage of energy (kcal) provided in the diet relative to nutrients—nutrients above the red line show the grain food’s contribution to nutrient density.

The percentage of total dietary intake of energy and nutrients provided from the cooked grains category can be seen in Figure 16. When considering nutrients to limit, the ready-to-eat cereals category provided 2.0% of sodium, 0.1% of total sugar, 0.6% of total fat and 0.4% of saturated fat in the daily diet. Cooked grains provided 1.5% of total energy, 1.3% of dietary fiber, 3.1% of dietary folate, 1.8% of iron, 2.2% of thiamin, 1.6% of niacin, 1.4% of magnesium, 1.2% of zinc and 1.1% of vitamin B6, with negligible contributions of vitamins A, D, B12, E, riboflavin and potassium.

### 3.5. Quick Breads and Bread Products: Sources of Energy and Nutrients

**Figure 21:**
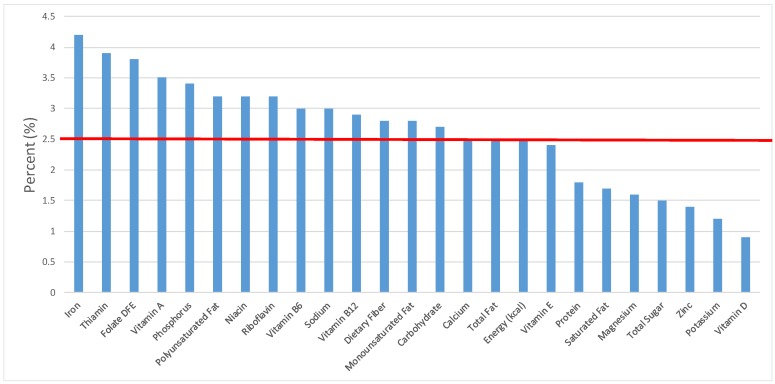
Quick breads and bread products as sources of energy and nutrients for children and adolescents 2–18 years old (*N* = 6109, daily intake data): DFE = Dietary Folate Equivalents; solid red line represents percentage of energy (kcal) provided in the diet relative to nutrients—nutrients above the red line show the grain food’s contribution to nutrient density.

The percentage of total dietary intake of energy and nutrients provided from quick breads and bread products can be seen in Figure 21. When considering nutrients to limit, the quick breads and bread products category provided 3.0% of sodium, 1.5% of total sugar, 2.5% of total fat and 1.7% of saturated fat in the daily diet. In addition, quick breads and bread products contributed 2.5% of total energy, 2.8% of dietary fiber, 3.8% of folate, 4.2% of iron, 3.9% of thiamin, 3.2% of niacin, 1.6% of magnesium, 1.4% of zinc, 3.0% of vitamin B6, 2.9% of vitamin B12, 3.5% of vitamin A, 3.2% of riboflavin and 2.4% of vitamin E, with negligible contributions of vitamins D.

### 3.6. Sweet Bakery Products: Sources of Energy and Nutrients

**Figure 26:**
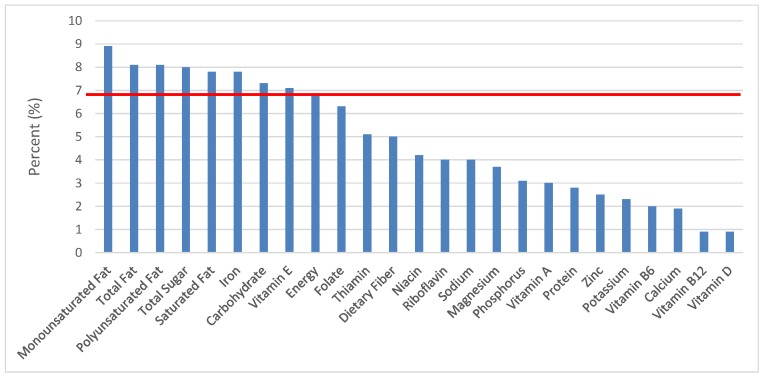
Sweet bakery products as sources of energy and nutrients for children and adolescents 2–18 years old (*N* = 6109, daily intake data): DFE = Dietary Folate Equivalents; solid red line represents percentage of energy (kcal) provided in the diet relative to nutrients—nutrients above the red line show the grain food’s contribution to nutrient density.

The percentage of total dietary intake of energy and nutrients provided from sweet bakery products can be seen in Figure 26. When considering nutrients to limit, the sweet bakery grains (i.e., cakes, cookies, pies, etc.) provided meaningful amounts for several nutrients to limit for 6.8% of total energy including, 8.1% of total fat, 7.8% of saturated fat and 8.0% of total sugar in the daily diet. In addition, sweet bakery grains contributed modestly to nutrients to encourage and had a smaller contribution to sodium compared with the percent of total energy: 5.0% of dietary fiber, 6.3% of folate, 7.8% of iron, 5.1% of thiamin, 4.2% of niacin, 4.0% of riboflavin and 7.1% of vitamin E, as well as 4.0% of sodium with negligible contributions of magnesium, zinc, potassium and vitamins A, B12 and B6.

## 4. Discussion

The present analysis determined the contributions of all grain foods and specific grain food groups for energy (calories) and nutrients to the diet of American children and adolescents and provides evidence that certain grain foods are meaningful contributors to several shortfall nutrients as identified by the 2015–2020 DGA. All grain products contributed 14% or 263 kcal per day in the total diet of children and adolescents. When considering nutrients to limit as outlined by dietary guidance, grain foods contributed 7.6% total fat, 5.4% saturated fat, 15.9% sodium and 8% total sugar. Similarly, when considering nutrients to encourage (i.e., shortfall nutrients), grain foods are meaningful contributors of nutrient density. Additionally, breads, rolls, tortillas and ready-to-eat cereals are meaningful contributors of dietary fiber, thiamin, folate, iron, zinc and niacin, while some grain foods alone provide minimal contributions to the diet, including sweet bakery products. Cumulatively, a variety of grain food groups consumed by American children and adolescents contribute to nutrient density in the total diet and should be encouraged as part of a healthy dietary pattern.

While the grain food category contributed energy, total fat, saturated fat, total sugar and sodium, grain foods also provided a substantial contribution for folate, iron and magnesium per day, all of which are under-consumed nutrients in U.S. children and adolescents. The examination of enriched and fortified foods on nutrient intakes using NHANES data in children and adolescents has previously shown that without fortification and enrichment, 20% of the U.S. population would fall below the EAR for iron, with this percentage being lowered to approximately 6% when including enriched and fortified foods. Likewise, the contribution of enriched and fortified foods to riboflavin and niacin showed a meaningful contribution of these foods in the diets of U.S. children and adolescents [17]. The most robust effects of enrichment and fortification practices were seen with thiamin and folic acid nutrients typically added to grain foods. Indeed, without enrichment and fortification, 50% of the U.S. population would have inadequate intakes compared with only 5% when enrichment and fortification is included in the analysis. Similarly, for folic acid, when not considering mandatory and discretionary enrichment and fortification, nearly 90% of Americans would have inadequate intake versus only 10% with enriched and fortified food consumption, thus showcasing the relevance of enrichment and fortification practices in reducing the percentage of the population below the EAR for nutrients routinely sourced from grains [17,24]. Similarly, our analyses showed that all grain foods are meaningful contributors of nutrients of public health concern as outlined by the 2015–2020 Dietary Guidelines for Americans, including dietary fiber and calcium. Our current analyses also assessed the sources of energy and nutrients from specific grain foods, of which included breads, rolls, tortillas and ready-to-eat cereals. While breads, rolls and tortillas contributed less than 10% of all sodium (7.7%), total fat (3.1%) and saturated fat (2.2%), breads, rolls and tortillas provided higher than 10% for dietary fiber (12%), folate (14.2%) and iron (11.7%). Similarly, ready-to-eat cereals provided minimal daily contributions of energy (3.3%), sodium (3.0%), total sugar (4.0%), total fat (1.2%) and saturated fat (0.9%), but meaningful contributions of folate (18.2%), iron (16.6%), thiamin (12.3%), vitamin B12 (13.0%), vitamin A (11.7%), niacin (12.5%) and zinc (10.3%). Thus, the nutrient contribution of whole and refined grain food products, including breads, rolls, tortillas and ready-to-eat cereals, can play a key role in helping American children and adolescents meet recommendations for under-consumed nutrients and dietary fiber, a nutrient of public health concern [4]. Our work is aligned with previously-reported data where researchers showed that while three of the top 10 sources of energy provided no nutritional value, the remaining sources of energy, including milk, beef, poultry, cheese and baked goods were significant contributors of nutrients of concern and other essential nutrients, and thus, eliminating these foods from food patterns could potentially have inadvertent effects on diet quality in the U.S. population [16]. While certain grain food products, including cooked grains, quick breads and bread products, provided minimal nutrient contributions, these grain categories also provided minimal energy density in the diet of children and adolescents, which is also an important aspect of current dietary guidance. Furthermore, these grain products tend to be consumed with nutrient-dense milk and milk products (i.e., cooked grains/quick breads and dairy products); thus, particular grain foods may serve as vehicles for nutrient-dense food group consumption in children and adolescents. Future research considering this notion would add value to the currently available literature.

While several key nutrients are under-consumed and falling short of recommendations in all U.S. children and adolescents, further research may need to focus on populations at greater risk for nutrient inadequacy. In a recent NHANES 2007–2010 analyses from our group [14], we identified significantly lower usual nutrient intakes in both non-Hispanic black children and adults when compared to their non-Hispanic white counterparts. Specifically in children, we observed that non-Hispanic black children had lower intakes of calcium, phosphorus, magnesium and vitamins A and D, in comparison to non-Hispanic white children. While nutrient inadequacy was apparent in children of all ethnicities examined, the prevalence of inadequate intake exceeded 25% of the population for calcium, phosphorus, magnesium and vitamins A, D and E in non-Hispanic black children relative to their white counterparts. Thus, specific populations may further benefit from including both whole and refined grain foods, in combination with other nutrient-dense foods (i.e., dairy, fruits, vegetables, lean protein) in their diet to help close gaps in under-consumed nutrients and nutrients of public health concern.

The current analyses have a limitation inherent in observational research. The results are dependent on self-reported dietary data for foods, which may involve study participants under- or over-estimating food consumption, leading to inaccuracies in energy and nutrient intakes. Data were also obtained using a 24-h dietary recall, which relies on study participant memory, and while validated methods are used to gather the data, recall information is subject to inaccuracies and bias from memory challenges and other potential measurement errors experienced in epidemiological investigations using large datasets [25]. Caution should be administered when comparing the current findings to previous studies, as food groupings can impact sources of energy and nutrient outcomes, specifically where there are differences in the level of aggregation (i.e., the number of food groups) or disaggregation methods used by researchers. A significant benefit of using NHANES data for the current analyses includes access to a large and nationally-representative dataset of children and adolescents in the U.S. and corresponding food and nutrient intake data.

## 5. Conclusions

Despite authoritative recommendations to increase specific nutrients in the U.S. population through food consumption, children and adolescents are falling short of reaching several nutrient recommendations. Our current analyses show that cumulatively, a variety of grain food groups consumed by American children and adolescents help contribute to nutrient density in the total diet. While grain foods as a category are contributors of energy, total fat and sodium in the U.S. diet, grain foods are also contributors of the 2015–2020 DGA under-consumed nutrients and nutrients of public health concern [4], including dietary fiber, folate, magnesium, calcium and iron. When considering sub-categories of grain foods, breads, rolls, tortillas and ready-to-eat cereals are meaningful contributors (i.e., ≥10% in the diet) of dietary fiber, thiamin, folate, iron, zinc and niacin to the American diet of children and adolescents. Encouraging certain grain food patterns in the diets of children and adolescents, including selecting a mix of enriched and fortified grains, may improve nutrient intakes and minimize gaps in shortfall nutrient intakes. In contrast, eliminating grains, including both whole and refined grains, from the diet may lead to unintended nutrient consequences. Therefore, dietary strategies that aim to stay within calorie needs and recommendations while monitoring nutrients to limit (i.e., added sugars, sodium and saturated fat) can benefit from the inclusion of several refined and whole grain food products in the diet. A variety of grain food patterns, including those recommended by dietary guidance and those that focus on enriched and fortified grain staples, can provide a nutrient-dense variety of foods for children and adolescents while simultaneously helping to meet authoritative nutrient recommendations in this population. Future research must consider repeating the current analysis in different U.S. populations, including adults, children and adolescents of different ethnicities and age groups.

## Figures and Tables

**Figure 2:**
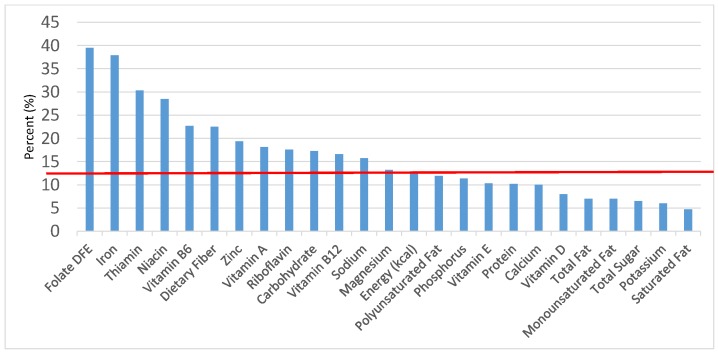
Grain foods sources of energy and nutrients for children 1–3 years old (*N* = 1423, daily intake data): DFE = Dietary Folate Equivalents; solid red line represents percentage of energy (kcal) provided in the diet relative to nutrients—nutrients above the red line show the grain food’s contribution to nutrient density.

**Figure 3:**
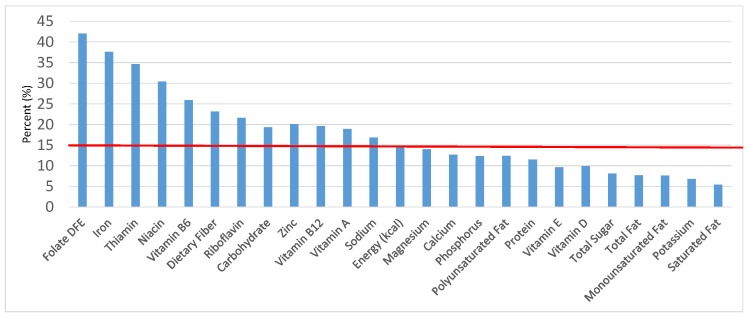
Grain foods sources of energy and nutrients for children 4–8 years old (*N* = 1917, daily intake data): DFE = Dietary Folate Equivalents; solid red line represents percentage of energy (kcal) provided in the diet relative to nutrients—nutrients above the red line show the grain food’s contribution to nutrient density.

**Figure 4:**
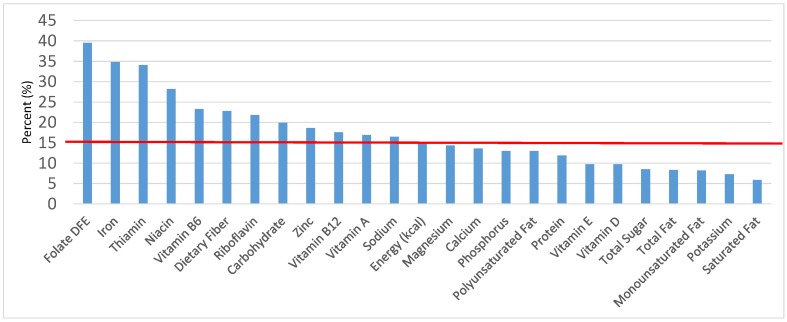
Grain foods sources of energy and nutrients for children and adolescents 9–13 years old (*N* = 1730, daily intake data): DFE = Dietary Folate Equivalents; solid red line represents percentage of energy (kcal) provided in the diet relative to nutrients—nutrients above the red line show the grain food’s contribution to nutrient density.

**Figure 5:**
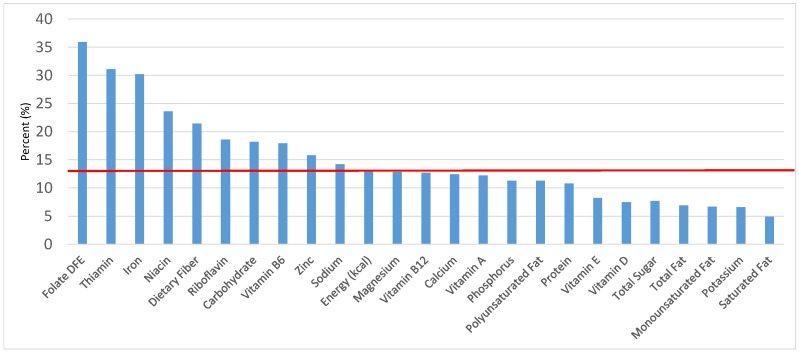
Grain foods sources of energy and nutrients for adolescents 14–18 years old (*N* = 1508, daily intake data): DFE = Dietary Folate Equivalents; solid red line represents percentage of energy (kcal) provided in the diet relative to nutrients—nutrients above the red line show the grain food’s contribution to nutrient density.

**Figure 7:**
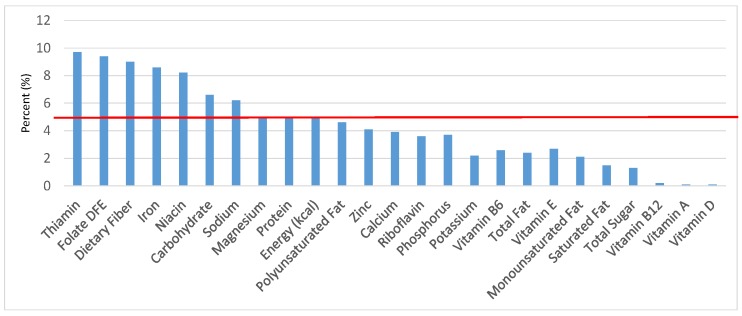
Breads, rolls and tortilla as sources of energy and nutrients for children 1–3 years old (*N* = 1423, daily intake data): DFE = Dietary Folate Equivalents; solid red line represents percentage of energy (kcal) provided in the diet relative to nutrients—nutrients above the red line show the grain food’s contribution to nutrient density.

**Figure 8:**
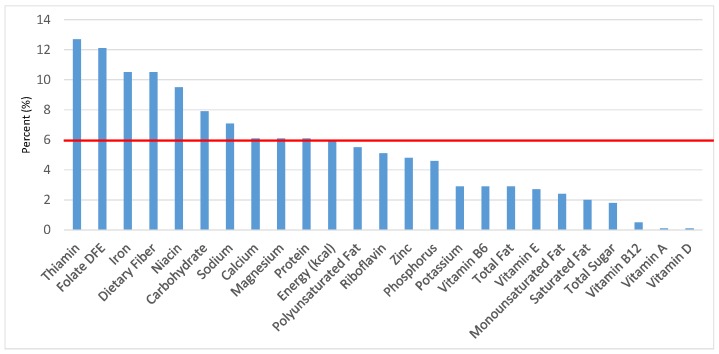
Breads, rolls and tortilla as sources of energy and nutrients for children 4–8 years old (*N* = 1917, daily intake data): DFE = Dietary Folate Equivalents; solid red line represents percentage of energy (kcal) provided in the diet relative to nutrients—nutrients above the red line show the grain food’s contribution to nutrient density.

**Figure 9:**
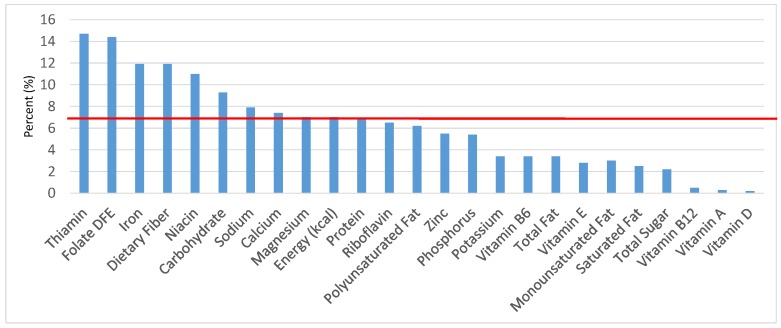
Breads, rolls and tortilla as sources of energy and nutrients for children and adolescents 9–13 years old (*N* = 1730, daily intake data): DFE = Dietary Folate Equivalents; solid red line represents percentage of energy (kcal) provided in the diet relative to nutrients—nutrients above the red line show the grain food’s contribution to nutrient density

**Figure 10:**
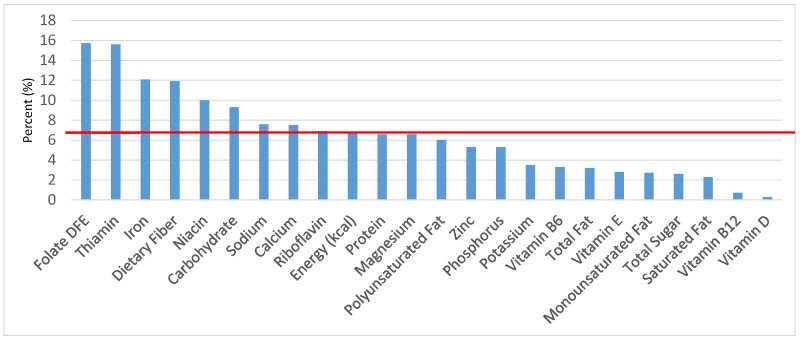
Breads, rolls and tortilla as sources of energy and nutrients for adolescents 14–18 years old (*N* = 1508, daily intake data): DFE = Dietary Folate Equivalents; solid red line represents percentage of energy (kcal) provided in the diet relative to nutrients—nutrients above the red line show the grain food’s contribution to nutrient density.

**Figure 12:**
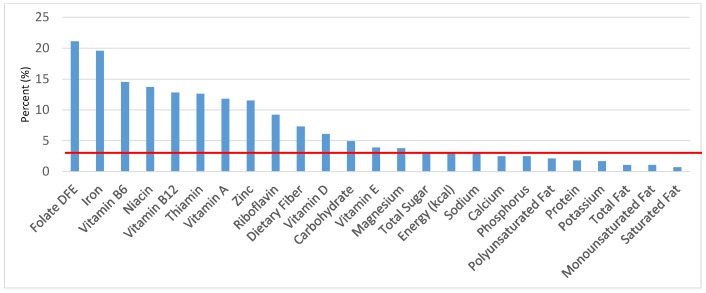
Ready-to-eat cereals as sources of energy and nutrients for children 1–3 years old (*N* = 1423, daily intake data): DFE = Dietary Folate Equivalents; solid red line represents percentage of energy (kcal) provided in the diet relative to nutrients—nutrients above the red line show the grain food’s contribution to nutrient density.

**Figure 13:**
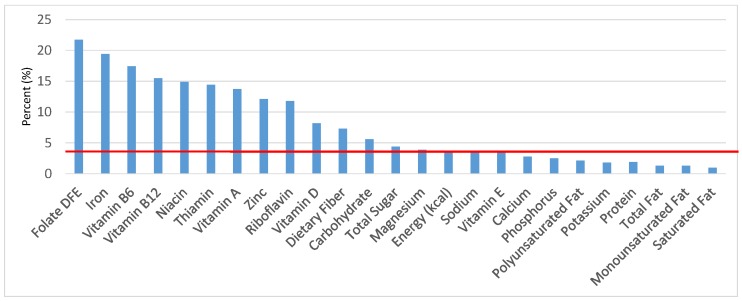
Ready-to-eat cereals as sources of energy and nutrients for children 4–8 years old (*N* = 1917, daily intake data): DFE = Dietary Folate Equivalents; solid red line represents percentage of energy (kcal) provided in the diet relative to nutrients—nutrients above the red line show the grain food’s contribution to nutrient density.

**Figure 14:**
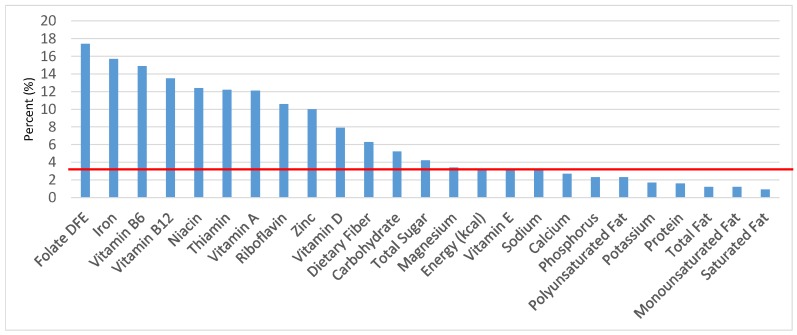
Ready-to-eat cereals as sources of energy and nutrients for children and adolescents 9–13 years old (*N* = 1730, daily intake data): DFE = Dietary Folate Equivalents; solid red line represents percentage of energy (kcal) provided in the diet relative to nutrients—nutrients above the red line show the grain food’s contribution to nutrient density.

**Figure 15:**
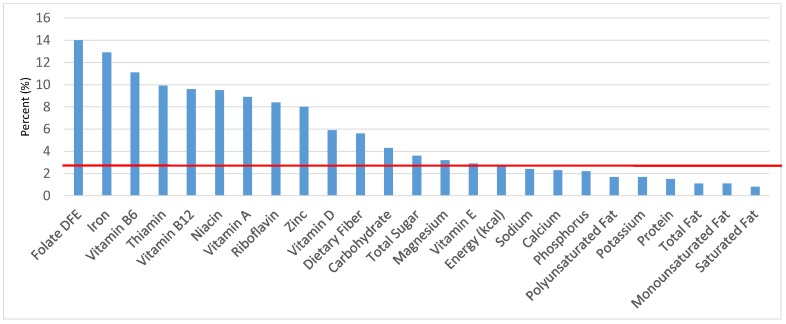
Ready-to-eat cereals as sources of energy and nutrients for adolescents 14–18 years old (*N* = 1508, daily intake data): DFE = Dietary Folate Equivalents; solid red line represents percentage of energy (kcal) provided in the diet relative to nutrients—nutrients above the red line show the grain food’s contribution to nutrient density.

**Figure 17:**
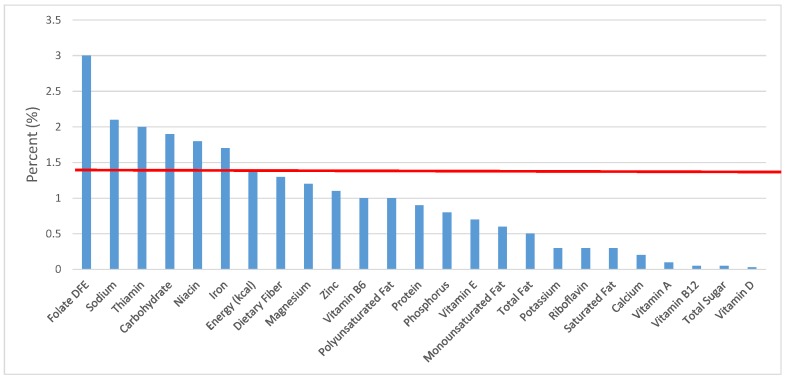
Cooked grains as sources of energy and nutrients for children 1–3 years old (*N* = 1423, daily intake data): DFE = Dietary Folate Equivalents; solid red line represents percentage of energy (kcal) provided in the diet relative to nutrients—nutrients above the red line show the grain food’s contribution to nutrient density.

**Figure 18:**
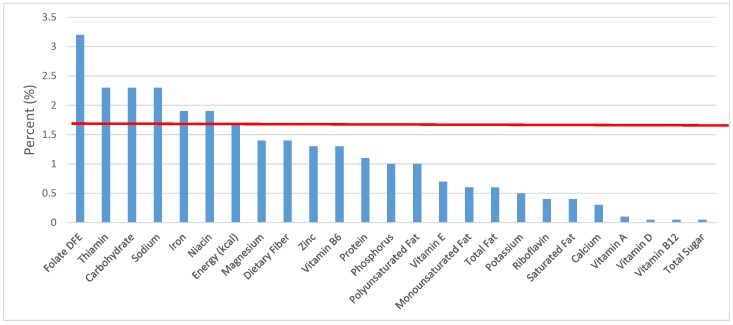
Cooked grains as sources of energy and nutrients for children 4–8 years old (*N* = 1917, daily intake data): DFE = Dietary Folate Equivalents; solid red line represents percentage of energy (kcal) provided in the diet relative to nutrients—nutrients above the red line show the grain food’s contribution to nutrient density.

**Figure 19:**
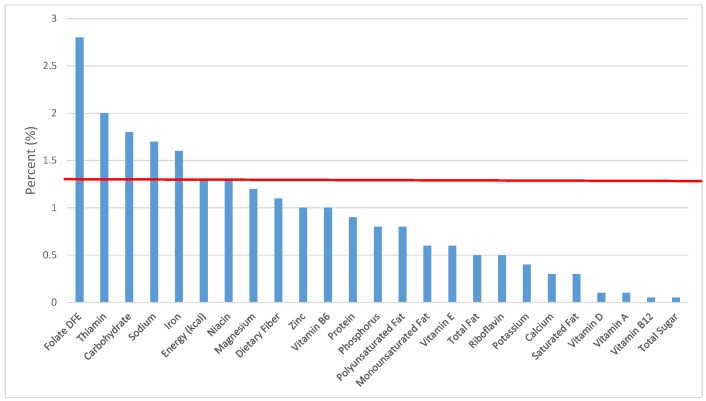
Cooked grains as sources of energy and nutrients for children and adolescents 9–13 years old (*N* = 1730, daily intake data): DFE = Dietary Folate Equivalents; solid red line represents percentage of energy (kcal) provided in the diet relative to nutrients—nutrients above the red line show the grain food’s contribution to nutrient density.

**Figure 20:**
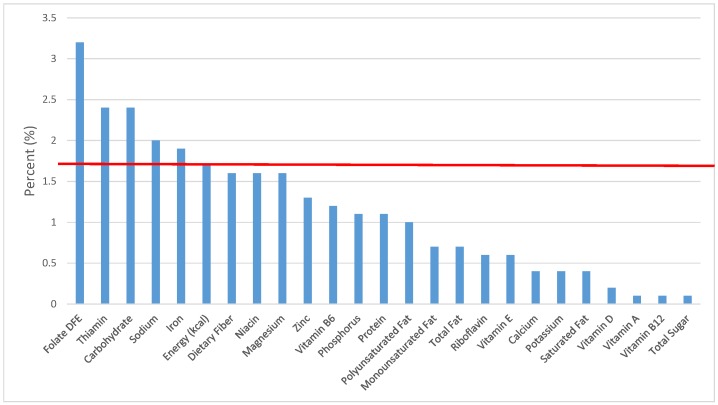
Cooked grains as sources of energy and nutrients for adolescents 14–18 years old (*N* = 1508, daily intake data): DFE = Dietary Folate Equivalents; solid red line represents percentage of energy (kcal) provided in the diet relative to nutrients—nutrients above the red line show the grain food’s contribution to nutrient density.

**Figure 22:**
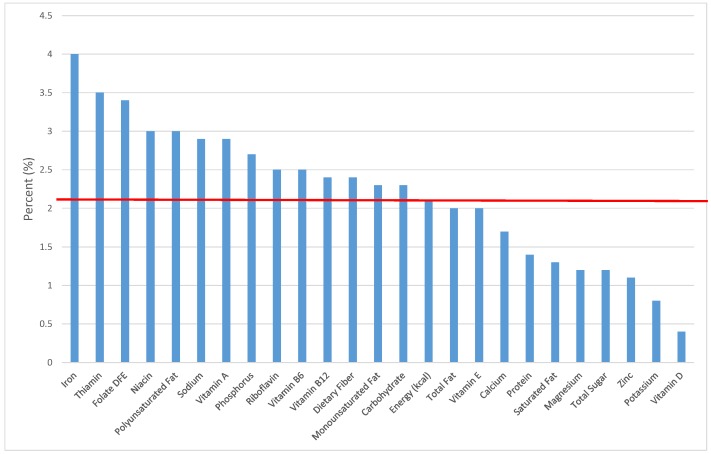
Quick breads and bread products as sources of energy and nutrients for children 1–3 years old (*N* = 1423, daily intake data): DFE = Dietary Folate Equivalents; solid red line represents percentage of energy (kcal) provided in the diet relative to nutrients—nutrients above the red line show the grain food’s contribution to nutrient density.

**Figure 23:**
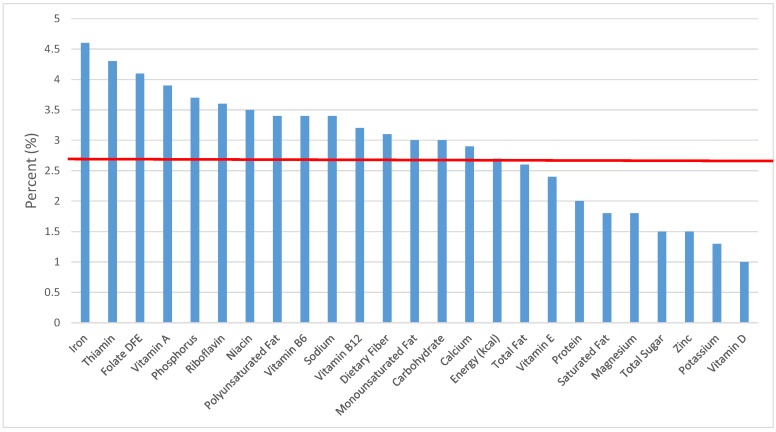
Quick breads and bread products as sources of energy and nutrients for children 4–8 years old (*N* = 1917, daily intake data): DFE = Dietary Folate Equivalents; solid red line represents percentage of energy (kcal) provided in the diet relative to nutrients—nutrients above the red line show the grain food’s contribution to nutrient density.

**Figure 24:**
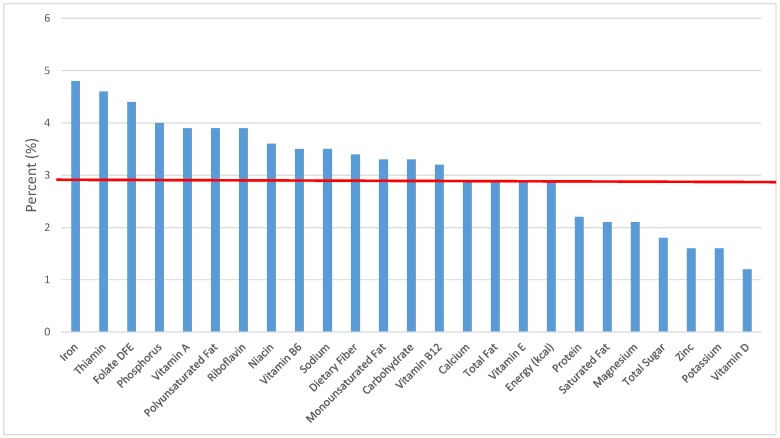
Quick breads and bread products as sources of energy and nutrients for children and adolescents 9–13 years old (*N* = 1730, daily intake data): DFE = Dietary Folate Equivalents; solid red line represents percentage of energy (kcal) provided in the diet relative to nutrients—nutrients above the red line show the grain food’s contribution to nutrient density.

**Figure 25:**
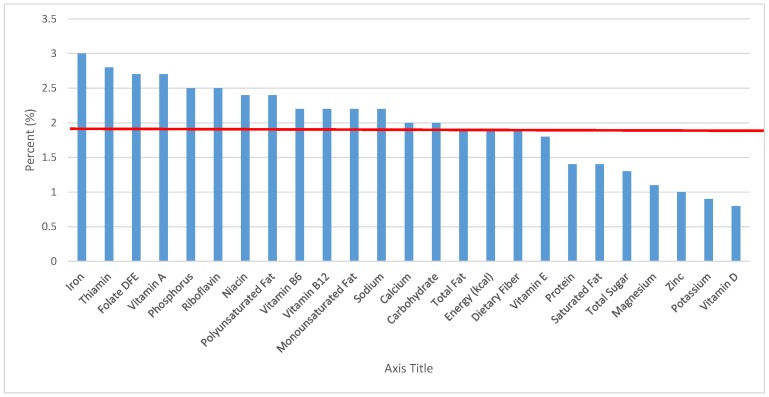
Quick breads and bread products as sources of energy and nutrients for adolescents 14–18 years old (*N* = 1508, daily intake data): DFE = Dietary Folate Equivalents; solid red line represents percentage of energy (kcal) provided in the diet relative to nutrients—nutrients above the red line show the grain food’s contribution to nutrient density.

**Figure 27:**
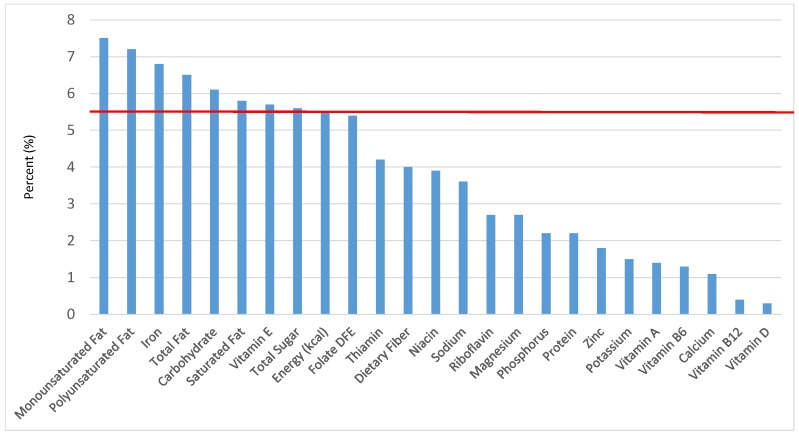
Sweet bakery products as sources of energy and nutrients for children 1–3 years old (*N* = 1423, daily intake data): DFE = Dietary Folate Equivalents; solid red line represents percentage of energy (kcal) provided in the diet relative to nutrients—nutrients above the red line show the grain food’s contribution to nutrient density.

**Figure 28:**
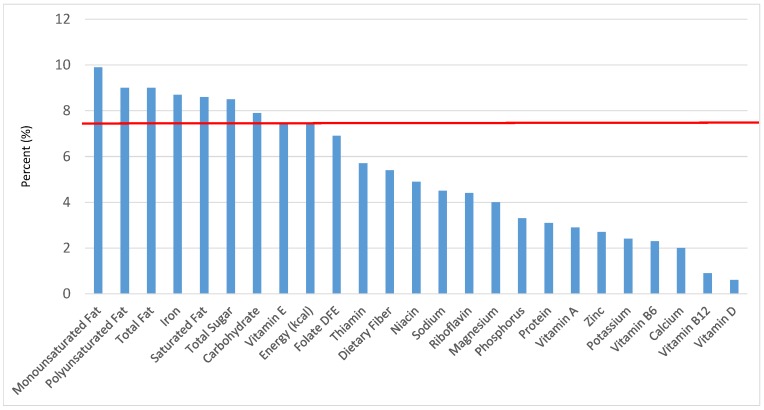
Sweet bakery products as sources of energy and nutrients for children 4–8 years old (*N* = 1917, daily intake data): DFE = Dietary Folate Equivalents; solid red line represents percentage of energy (kcal) provided in the diet relative to nutrients—nutrients above the red line show the grain food’s contribution to nutrient density.

**Figure 29:**
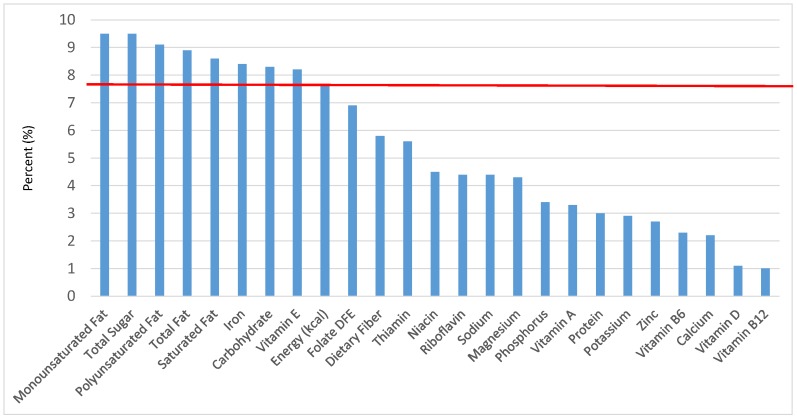
Sweet bakery products as sources of energy and nutrients for children and adolescents 9–13 years old (*N* = 1730, daily intake data): DFE = Dietary Folate Equivalents; solid red line represents percentage of energy (kcal) provided in the diet relative to nutrients—nutrients above the red line show the grain food’s contribution to nutrient density.

**Figure 30:**
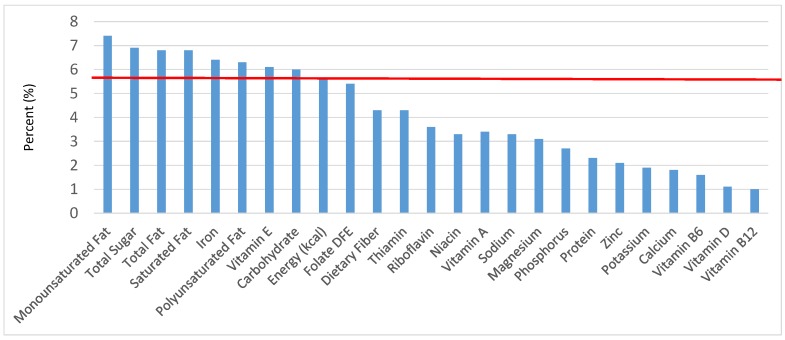
Sweet bakery products as sources of energy and nutrients for adolescents 14–18 years old (*N* = 1508, daily intake data): DFE = Dietary Folate Equivalents; solid red line represents percentage of energy (kcal) provided in the diet relative to nutrients—nutrients above the red line show the grain food’s contribution to nutrient density.

**Table 1:** Grain categories identified from National Health and Nutrition Examination Survey 2009–2012 in children and adolescents (2–18 years old; *N* = 6109) by mean ± standard error (SE) gram weight and percent weight (SE) in total diet.

Grain Food Group Category	Mean Weight (g) Consumption (SE)	Percent Weight Consumption in the Total Diet
All Foods	2208 ± 34	100% ± 0
All Grains	104 ± 2	5.18 ± 0.11
Breads, Rolls and Tortillas	43.6 ± 1.3	2.16 ± 0.05
Ready-to-Eat Cereals	15.3 ± 0.6	0.79 ± 0.03
Cooked Grains	20.0 ± 1.8	0.96 ± 0.08
Quick Breads and Bread Products	16.9 ± 1.0	0.88 ± 0.06
Sweet Bakery Products	34.4 ± 1.1	1.73 ± 0.06
